# Prevalence and factors associated with incomplete immunization among children aged 12 to 35 months in Eswatini: analysis of the Eswatini multiple indicator cluster survey

**DOI:** 10.11604/pamj.2023.45.51.38643

**Published:** 2023-05-23

**Authors:** Thatho Nolwazi Dlamini, Lekha Divakara Bhat, Naveen Kumar Kodali, Neena Elezebeth Philip

**Affiliations:** 1 Department of Epidemiology and Public Health, Central University of Tamil Nadu, Tamil Nadu, India

**Keywords:** Incomplete immunization, prevalence, risk factors, Eswatini multiple indicator cluster survey

## Abstract

**Introduction:**

incomplete childhood immunization is a significant public health challenge as children continue to succumb to vaccine-preventable diseases in most developing countries. Studies on childhood immunization conducted in Eswatini are sparse. Therefore, the present study assessed the prevalence of incomplete childhood immunization in Eswatini and further explored associated factors among children aged 12 to 35 months.

**Methods:**

using data from Eswatini multiple indicator cluster survey 5 (EMICS5), a cross-sectional analysis with 978 children aged 12 to 35 months was conducted. This is the latest available data in the public domain. The survey was conducted from July 2014 to October 2014. The primary outcome variable was incomplete immunization. Univariate and multivariate logistic regressions were used to examine the association between selected variables and incomplete immunization.

**Results:**

the mean age of the children was 23.45±6.92 months, 50.2% were boys, and 74.1% lived in rural areas. The prevalence of incomplete immunization was 31.5%. Increased child´s age, being a girl, increased caregiver´s age, and increased number of children under-five years in the household and residing in the Manzini or Hhohho region were significantly associated with incomplete immunization.

**Conclusion:**

the EMICS 5 revealed a high prevalence of incomplete immunization in Eswatini. Health promotion activities such as empowering women and caregivers of children through health education about child health should be emphasized. Where feasible, outreach services and door-to-door immunization should be strengthened to improve immunization coverage in the country and cover dropouts.

## Introduction

Childhood immunization is an important public health intervention that has significantly contributed to improving child health indicators such as under-five mortality rate [1]. Despite the availability of vaccines, incomplete childhood immunization is a reality, especially in many developing countries. For example, in 2020, of the 17.1 million children who did not receive an initial dose of the diphtheria, pertussis, and tetanus (DPT) vaccine, 60% of them were from ten developing countries [2]. The World Health Organization (WHO) estimates that one in five children in Africa do not receive all essential vaccines [3]. As a result, Africa accounts for 58% of the global under-five mortality rate annually from vaccine-preventable diseases (VPDs) [3]. High burden of child mortalities is observed in sub-Saharan Africa, contributing 54% to the global child mortality rate [4]. Vaccine-preventable diseases constituted 21•7% of under-five mortalities in 2019 [5]. Immunization remains one of the safe and cost-effective public health interventions in preventing premature mortalities and controlling epidemics [6,7]. Eswatini is a relatively small country in sub-Saharan Africa with a population of around 1.2 million and a population density of about 67 inhabitants per square kilometer as of April 2023 [8]. Noticeable differences in the population density exist within the four administrative regions of the country (Hhohho (89), Manzini (87), Shiselweni (54), and Lubombo (36) inhabitants per square kilometer) [9]. Seventy-six percent of the entire population [10] and 80% of children between zero and four years live in rural areas [9]. The current trends in the urban-rural population ratio indicate that a substantial proportion of the population resides in the rural areas in all the regions of Eswatini (Hhohho 30, Lubombo 16.3, Shiselweni 7.5) except for Manzini, which represents around 66 urban residents for 100 persons living in rural areas [9]. Eswatini´s development landscape is skewed, with higher inequality, unemployment and poverty among the rural population. Eswatini is classified as a lower-middle-income country, with a gross domestic product of $3,034 per capita [11], and is ineligible for Gavi support [12]. The country's universal health service coverage index increased from 46% in 2005 to 58% in 2019, growing at an average annual rate of 6.05% [13]. The Eswatini healthcare system is structured as a decentralized model, with health services being provided by both public and private providers. As per the 2017 service availability and readiness assessment (SARA) report, most (n=130) facilities are in Manzini, followed by Hhohho (n=100), Lubombo (n=52) and Shiselweni (n=45) [14]. The expanded program of immunization (EPI) in Eswatini forms part of child survival program and receives full government support [15]. The immunization program is delivered through a network of health facilities and outreach sites [15]. However, there is no readily available data on the proportion of immunizations provided through static sites versus outreach sites.

Even though childhood immunizations are provided freely throughout Eswatini, the proportion of children aged 12-23 months and 24-35 months receiving the full schedule of vaccinations in the country is around 70.7% and 65%, respectively [16]. This immunization coverage is way below the Global Vaccine Action Plan target goal of 90% vaccine coverage at the national level [17]. The incompleteness in childhood immunization in Eswatini has contributed to occasional outbreaks of VPDs in the country, such as the 2019 cases of diarrhoea attributed to the rotavirus [18]. Alarmingly, 53.4 per 1000 live birth infants in Eswatini did not survive past the first year of life in 2014 [19]; and a further 52.6 children per 1000 live births died before celebrating their fifth birthday in 2021 [20]. At this rate, Eswatini is lagging behind the 2030 sustainable development goal (SDG), aiming to reduce under-five mortality to less than 25 in each country [21]. Full childhood immunization coverage has been proven to be one of the basic public health interventions to achieve the ambitious SDGs [22]. The available literature has revealed that there are currently no known vaccine-resistant groups in Eswatini. Studies conducted in countries with limited resources have identified sociodemographic factors such as maternal age, parental education, exposure to media, parity, maternal health-seeking behavior, place of delivery, family wealth index [23-25], community poverty status, place of residence, and literacy rate in the community [24], as some of the major contributing factors leading to incomplete childhood immunization. Data on the prevalence and factors associated with incomplete childhood immunization in Eswatini is sparse. Understanding which group of children in terms of cultural, social, and regional factors end up with incomplete immunization is crucial to develop evidence-based public health intervention so that national childhood immunization targets can be achieved. Therefore, the present study aimed to explore the pattern and factors associated with incomplete immunization coverage among children aged 12 to 35 months in Eswatini.

## Methods

**Data source, study design, and setting:** this cross-sectional study utilized secondary data from the 2014 Eswatini multiple indicator cluster survey 5 (EMICS 5) [16]. The EMICS 5 is the latest available data in the public domain. The multiple indicator cluster survey (MICS) is a nationally representative and cross-sectional household survey conducted every three to five years in developing countries to monitor key health indicators of men, women, and children [16].

**Data collection:** the EMICS 5 data were collected from July 2014 to October 2014 by interviewers specifically trained for 19 days in July 2014. Data was collected using four types of questionnaires during the EMICS 5: i) a household questionnaire to collect data on usual household residents and household characteristics; ii) an individual men´s questionnaire administered in every third household to male members of the household 15 to 49 years old; iii) individual women's questionnaire administered to female members of the household 15 to 49 years old; and iv) an individual questionnaire administered to mothers or caregivers of all children under-five years living in the household. The present study mainly utilized data from the individual questionnaires administrated to mothers or caretakers of children under-five years. During data collection, a multi-stage sampling approach was applied in the EMICS 5. The four administrative regions of Eswatini were stratified into rural and urban to form eight strata. A total of 347 enumeration areas, 5211 households, and 1118 children aged 12 to 35 months were surveyed [16].

**Eligibility criteria:** children between 12 and 35 months whose immunization-related data were collected during the EMICS 5 were eligible for this analysis. We excluded children under 12 months and those above 35 months because immunization-related data were not collected during the EMICS 5. Children with missing values (12.5%) were also excluded. This study then focused exclusively on 978 children aged 12 to 35 months.

### Study variables

**Outcome variable:** incomplete immunization was our outcome variable. The outcome variable was calculated using the 2014 Eswatini immunization schedule available at the time of data collection (Table 1). For children aged 12 to 23 months, the outcome variable was computed using nine doses of four vaccines: Bacille Calmette-Guérin (BCG) (1 dose), oral poliovirus vaccine (OPV) (4 doses), DPT (3 doses), and Measles (1 dose) by one year. For children aged 24 to 35 months, the outcome variable was estimated using eleven doses of four vaccines: BCG (1 dose), OPV (5 doses), DPT (3 doses), and Measles (2 doses) by 24 months. Pneumococcal conjugate vaccine (PCV13) was omitted when calculating the outcome variable because it was introduced into the immunization schedule three months before the data collection in the EMICS 5. Incomplete immunization was defined as a child who missed any of the recommended vaccine doses by either one year (for children aged 12 to 23 months) or two years (for children aged 24 to 35 months). However, since no variable in the dataset represented complete or incomplete immunization, a composite binary variable was computed where; 1) was coded for incomplete immunization and (0) for complete immunization (Table 1).

**Table 1:** Eswatini childhood immunization schedule (2014)

Age	Antigen
Birth	BCG + OPV0
6 weeks	OPV1+DPT1+ PCV13;1
10 weeks	OPV2+DPT2+ PCV13;2
14 weeks	OPV3+DPT3+ PCV13;3
9 months	Measles 1
18 months	OPV4 + Measles 2

**Predictor variables:** from the literature reviewed for the study and also considering the variables which were included during EMICS 5, the following independent variables were considered during the current analysis: child's age, gender, caregivers´ education, caregiver´s age, household size, number of children under-five in the household, household head´s gender, media exposure (access to television or radio whereby a yes or no type of question was asked), wealth index, region, and location of residence.

**Statistical analysis:** data were analyzed using Statistical package for the social sciences (SPSS) version 25. Sample weights were applied throughout the analyses to adjust for the complex sampling design of EMICS 5. Missing data were handled using the complete case analysis approach. Proportions were calculated for categorical variables and Pearson chi-square was used in the bivariate analysis to test the association between the categorical variables. The value '1' was allocated to the dependent dichotomous variable if the child had incomplete immunization and the value '0' if the child had complete immunization. Univariate logistic regression analysis was carried out to obtain the unadjusted odds' ratio (UOR) of predictors on the outcome variable, and those predictors whose values were found to have p<0.25 were entered into the multivariate logistic regression model to assess and derive the adjusted odds' ratio (AOR) on the outcome variable. A p<0.05 at a 95% confidence interval was considered statistically significant during the multivariate analysis.

**Ethical consideration:** the Eswatini Central statistics office ensured ethical compliance during the implementation of the 2014 EMICS, including obtaining ethical approval from the Eswatini Health and Human Research Review Board. The researcher had no special access privileges to the dataset, the data is publicly available, and other researchers can freely access it from the UNICEF website [26]. The participants were anonymous, and no attempt was made to identify them. The data set was used only for the current study purposes.

## Results

**Background characteristics:** there were 1118 children aged 12 to 35 months in the survey. Of these, 140 were excluded due to missing values. Sample weights were then applied. The weighted sample was 978 children aged 12 to 35 months with a mean age of 23.45 ± 6.92 months. Children between 24 and 35 months accounted for 51% (499/978) of the total sample. There were slightly more boys, 50.2% (490/978), than girls. Most caregivers, 28.9% (283/978), were 21 to 25 years old and 33.6% (329/978) had high school or tertiary education. Most of the children, 50.9% (498/978), were from female-headed households. A majority of the households, 75.7% (740/978), had access to mass media. Most households, 67.1% (656/978), had five or more family members, and 53.8% (526/978) of the households had one under-five child. The wealth quintile ranged from 17.6% (172/978) for rich households to 24.1% (236/978) for poor households. A significant proportion of the children (74.1%) were from rural areas. A higher percent (39.4%) of the children were from the Manzini region, and the Shiselweni region contributed the least proportion (18.4%) (Table 2).

**Table 2:** weighted prevalence of incomplete immunization by sociodemographic variables, Eswatini multiple indicator cluster survey 2014

Variable	Incomplete childhood immunization		Total	
	No n (%)	Yes n (%)	N (%)	p-value
**Child’s age**				
12-23 months	362 (75.6%)	117 (24.4%)	479 (49.0%)	<0.001
24 – 35 months	308 (61.7%)	191 (38.3%)	499 (51.0%)	
**Child’s gender**				
Boys	350 (71.4%)	140 (28.6%)	490 (50.2%)	0.063
Girls	320 (65.7%)	167 (34.3%)	487 (49.8%)	
**Caregiver's age**				
16-20	87 (82.9%)	18 (17.1%)	105 (10.7%)	0.005
21-25	182 (64.3%)	101 (35.7%)	283 (28.9%)	
26-30	169 (70.7%)	70 (29.3%)	239 (24.4%)	
31-35	114 (65.9%)	59 (34.1%)	173 (17.7%)	
36-40	69 (71.9%)	27 (28.1%)	96 9.8%)	
41-45	32 (65.3%)	17 (34.7%)	49 (5.0%)	
46-50	17 (51.5%)	16 (48.5%)	33 (3.4%)	
**Caregiver’s education**				
None or Primary	214 (65.2%)	114 (34.8%)	328 (33.5%)	0.098
Secondary	234 (72.9%)	87 (27.1%	321 (32.9%)	
High school or tertiary	222 (67.5%)	107 (32.5%)	329 (33.6%)	
**Sex of household head**				
Male	324 (67.5%)	156 (32.5%)	480 (49.1%)	0.536
Female	345 (69.4%)	152 (30.6%)	497 (50.9%)	
**Mass Media**				
Yes	505 (68.2%)	235 (31.8%)	740 (75.7%)	0.81
No	165 (69.3%)	73 (30.7%)	238 (24.3%)	
**Household size**				
Four or less	229 (71.3%)	92 (28.7%)	321 (32.9%)	0.212
Five or more	441 (67.2%)	215 (32.8%)	656 (67.1%)	
**Number of children under-five**				
1	379 (72.1%)	147 (27.9%)	526 (53.8%)	0.008
2-3	262 (66.0%)	135 (34.0%)	397 (40.6%)	
4 or more	29 (53.7%)	25 (46.3%)	54 (5.5%)	
**Residence location**				
Urban	171 (67.6%)	82 (32.4%)	253 (25.9%)	0.753
Rural	499 (68.8%)	226 (31.2%)	725 (74.1%)	
**Residential region**				
Hhohho	135 (62.5%)	81 (37.5%)	216 (22.1%)	0.019
Manzini	130 (72.2%)	50 (27.8%)	180 (18.4%)	
Lubombo	148 (75.5%)	48 (24.5%)	196 (20.1%)	
Manzini	256 (66.5%)	129 (33.5%)	385 (39.4%)	
**Wealth index**				
Poorest	146 (70.9%)	60 (29.1%)	206 (21.1%)	0.439
Poor	155 (65.7%)	81 (34.3%)	236 (24.1%)	
Middle	124 (66.3%)	63 (33.7%)	187 (19.1%)	
Rich	126 (73.3%)	46 (26.7%)	172 (17.6%)	
Richest	119 (67.2%)	58 (32.8%)	177 (18.1%)	
**Total**	**670 (68.5%)**	**308 (31.5%)**	**978 (100%)**	

**Prevalence of incomplete childhood immunization in Eswatini:** in this study, 31.5% (308/978) of the children had not received one or more of the EPI's recommended vaccines at the time of the survey. Incomplete immunization ranged from 37.5% in the Hhohho region to 24.5% in the Lubombo region. Within the area of residence, incomplete childhood immunization was slightly higher in urban areas than in rural areas (Table 2).

**Factors associated with incomplete immunization in Eswatini:** after controlling for covariates through a multiple logistic regression model, children aged 24 to 35 months (AOR=1.986, p<0.001) were more likely to be incompletely immunized than those aged 12 to 23 months. The odds of incomplete immunization were 1.306 times higher among girls than boys (p=0.053). Children whose caregiver was 21 to 25 years (p= 0.003), 26 - 30 years (p=0.039), 31 to 35 years (p=0.005), 41 to 45 years (p=0.055), and 46 to 50 years (p=0.001) were 2.469, 1.903, 2.508, 2.225, and 4.611 times, respectively, more likely to be incompletely immunized than children from caregivers aged 16 to 20 years. Children from households with 2 to 3 under-five children (p=0.041) and those from households with four or more under-five children (p=0.001) were 1.398 and 2.959 times more likely to be incompletely immunized. Children from the Hhohho region (p<0.001) and Manzini region (p=0.028) were 2.235 and 1.628 times, respectively, more likely to be incompletely immunized than those from the Lubombo region (Table 3).

**Table 3:** association between incomplete childhood immunization and sociodemographic variables, Eswatini multiple indicator cluster survey 5, 2014

			Multivariate analysis		
Variable	UOR	p-value	AOR	95% CI	p-value
**Child’s age**					
12-23 months	1	-	1	-	
24-35 months	1.933	<0.001	1.986	1.484 – 2.657	<0.001**
**Child’s gender**					
Boys	1	-	1	-	-
Girls	1.306	0.053	1.306	0.996 – 1.712	0.053*
**Caregiver’s age**					
16-20	1	-	1	-	-
21-25	2.735	<0.001	2.469	1.363 – 4.471	0.003**
26-30	2.053	0.015	1.903	1.031 – 3.511	0.039**
31-35	2.532	0.002	2.508	1.318 – 4.770	0.005**
36-40	1.938	0.056	1.725	0.854 – 3.485	0.128
41-45	2.714	0.12	2.225	0.983 – 5.035	0.055*
46-50	4.637	<0.001	4.611	1.884 – 11.287	0.001**
**Caregiver’s education**					
None or primary	1.097	0.574	1.095	0.719 – 1.668	0.671
Secondary	0.75	0.119	0.752	0.514 – 1.100	0.142
High school/tertiary	1	-	1	-	-
**Sex of household head**					
Male	1.098	0.499	-	-	-
Female	1	-	-	-	-
**Mass media**					
Yes	1	-	-	-	-
No	0.946	0.732	-	-	-
**Household size**					
4 or less	1	-	1	-	-
5 or more	1.212	0.196	1.081	0.754 – 1.551	0.671
**Number of children under-five**					
1	1	-	1	-	-
2-3	1.331	0.046	1.398	1.014 – 1.926	0.041**
4 or more	2.211	0.006	2.959	1.556 – 5.626	0.001**
**Residence location**					
Urban	1.053	0.741	-	-	-
Rural	1	-	-	-	-
**Residential region**					
Hhohho	1.847	0.005	2.235	1.404 – 3.557	0.001**
Shiselweni	1.174	0.494	1.218	0.742 – 2.000	0.436
Lubombo	1	-	1	-	-
Manzini	1.545	0.028	1.628	1.053 – 2.516	0.028**
**Wealth index**					
Poorest	1	-	1	-	-
Poor	1.266	0.252	1.266	0.846 – 1.896	0.252
Middle	1.235	0.334	1.235	0.805 – 1.893	0.334
Rich	0.897	0.636	0.897	0.571 – 1.408	0.636
Richest	1.187	0.44	1.187	0.768 – 1.834	0.44

** statistically significant at p<0.05; * significant at p<0.10

## Discussion

Our study is one among the first studies on the prevalence of incomplete immunization and factors associated with incomplete immunization among children aged 12 to 35 months in Eswatini. The present study revealed a 31.5% prevalence of incomplete immunization in Eswatini. A high prevalence of incomplete immunization was also reported in South Africa (40.8%) in a cross-sectional study utilizing secondary data from the South African Demographic and Health Survey [27] and also in Togo (36.3%) in a cross-sectional study using multiple indicator cluster survey data [28]. In the present study, predictors of incomplete immunization were the region of residence, child's age, gender, caregiver's age, and the number of children under-five years in the household. In agreement with studies conducted in Ethiopia [29] and South Africa [27] this study has also revealed regional variations within the country in the prevalence of incomplete immunization. Children living in two regions of Eswatini, Hhohho and Manzini, were more likely to be incompletely immunized compared to those in the Lubombo region. These variations in vaccine coverage can be attributed to factors such as vaccine stock-outs and long waiting times, as in the study from South Africa [27]. Even though Manzini has the most public/ private healthcare facilities [14], a majority of them are concentrated in urban areas and sparse in rural areas. In addition, Manzini also has the most specialized clinics of all the other regions in the country. However, these specialised clinics do not provide childhood immunization services. This could explain the low immunization rates in the region. The findings from this study also highlight that children aged 24 to 35 months are more likely to be incompletely immunized. These findings correlate with those reported in a descriptive analysis conducted in South Africa [30]. The probable explanation for the high chances of incomplete immunization as the child's age increases could be due to the busy work schedules of caregivers. According to the Eswatini Regulation of Wages Order, female employees are entitled to 12 weeks of maternity leave [31]. Mothers/caregivers utilize this time for child-care, including childhood immunization. However, for vaccines to be received later, for example, at 18 months, the parents tend to be occupied with work schedules. In addition, girls had higher chances of incomplete immunization than boys in this study. The findings from this study do not align with the study that revealed that there is no child gender preference in Eswatini [32]. Furthermore, contrasting evidence has been reported in Ghana, where complete immunization coverage was reported to favour the girl child [33].

Moreover, this study revealed that the chances of incomplete childhood immunizations increased with maternal or caregiver´s age. Young mothers tend to immunize their children better than older mothers in this study. Studies have associated young mothers/caregivers with increased utilization of health care services, thus ultimately improving childhood immunization among children from this age group [34]. Evidence from Ethiopia also indicates that an increased mother/caregiver's age increases the odds of timely immunization [35]. Contrasting evidence has been reported in another study conducted in Ethiopia, where incomplete immunization was primarily associated with young mothers less than 20 years [36]. In addition, the findings of this study have revealed that the chances of incomplete immunization increase as the number of under-five children increases in the household. Similar results were reported in western Kenya [37]. This study found that place of residence (urban vs rural), caretaker educational status, and wealth status of the family did not have any association with the child's complete vaccination status. Similar findings have been reported in Ethiopia [38]. The findings from this study might imply an equitable distribution of childhood immunization sites in the urban and rural areas of Eswatini. However, spatial analysis of the distribution of childhood immunization sites in Eswatini between urban and rural residences should be done to provide tangible scientific evidence. Regarding caregiver´s education, similar findings have also been reported in studies conducted in Mozambique [39] and Ghana [33], where maternal/caregiver education level did not have an impact on the child´s immunization status. The findings from this study are incongruent with the results from other studies conducted in sub-Saharan Africa, where complete immunization was found to be pro-rich [24,25,29,40-42]. This could be because immunizations are provided free of cost in Eswatini.

**Strengths and limitations of the study:** this study has provided insight on the prevalence of incomplete childhood immunization in Eswatini and context-specific factors associated with the incomplete childhood immunization. This study utilized a nationally representative household survey dataset with a relatively large sample size, and sample weights were applied for all analyses, which enhanced the study's external validity, thus enabling the generalization of the findings. This study updates the evidence on the use of childhood immunization in Eswatini following the 2015 study that utilized the 2006-2007 Swaziland Demographic and Health Survey dataset [43]. To our knowledge, this is the first study to assess the prevalence of incomplete immunization and associated factors in Eswatini. Despite the above-mentioned strengths, the current study had some limitations. Firstly, as per the nature of secondary data analysis, some known predictors of incomplete immunization (such as distance to the health facility and the child's caregiver's health autonomy) were not available in the EMICS 5 dataset and therefore could not be analysed in the present study, which may have biased the results of the current study. The cross-sectional nature of the EMICS 5 limits the ability to establish cause-effect relationships between the explanatory variables and the outcome variables used in this study.

## Conclusion

With 31.5% of children between 12 and 35 months incompletely immunized in Eswatini, incomplete childhood immunization coverage remains high in the study sample. The main predictors of incomplete childhood immunization in Eswatini include increased child´s age, being a girl, increased caregiver´s age, increased number of children under-five years in the household, and residing in the Manzini or Hhohho region. Therefore, the authors recommend that during program planning, the child health program should ensure the equity in distribution of childhood immunization services to the different administrative regions of Eswatini. In addition, health promotion activities such as empowering children´s caregivers with knowledge on child health must be strengthened. Where feasible, outreach and door-to-door immunization of children need to be strengthened to improve immunization coverage in the country, which may also cover the dropouts. In addition, there is a need for health system strengthening, such as having flexible immunization hours and days, for example, ensuring that immunization services are provided throughout the day and during weekends. Such initiatives may help reduce incomplete immunization in Eswatini as most health facilities in the country provide childhood immunization services only in the morning on weekdays, whereas most parents are either at work or school during those periods. There is also a need for qualitative studies to explore further cultural, belief oriented specific reasons for incomplete childhood immunization in Eswatini.

### What is known about this topic

- *Childhood immunization is an essential public health intervention that helps improve child health indicators;*
- *Childhood immunization coverage and the factors influencing the coverage vary in different settings*.

### What this study adds

- *Incomplete childhood immunization in Eswatini is high- 31.5%;*
- *Factors contributing to incomplete childhood immunization in Eswatini include increased child’s age, being a girl child, increased caregiver’s age, increased number of children under-five years in the household and residing in the Manzini or Hhohho region of the country*.
